# Ambulatory Blood Pressure and Subclinical Cardiovascular Disease in Patients with Congenital Adrenal Hyperplasia: A Preliminary Report

**DOI:** 10.4274/jcrpe.1658

**Published:** 2015-03-05

**Authors:** Nesibe Akyürek, Mehmet Emre Atabek, Beray Selver Eklioğlu, Hayrullah Alp

**Affiliations:** 1 Necmettin Erbakan University Faculty of Medicine, Department of Pediatric Endocrinology and Diabetes, Konya, Turkey; 2 Necmettin Erbakan University Faculty of Medicine, Department of Pediatric Cardiology, Konya, Turkey

**Keywords:** Subclinical cardiovascular disease, congenital adrenal hyperplasia, ambulatory blood pressure

## Abstract

**Objective:**

There is an increased risk of cardiovascular morbidity in children and adolescents with classical congenital adrenal hyperplasia (CAH), presumably associated with obesity, hypertension, impaired glucose tolerance and dyslipidemia. This study was designed to evaluate the metabolic and cardiovascular profile of a group of children with classical CAH from the perspective of cardiovascular risk.

**Methods:**

Twenty-five CAH patients and 25 healthy controls were included in the study. Metabolic and anthropometric parameters were investigated and compared in these two groups.

**Results:**

Subjects in the CAH group were shorter than the controls (p=0.001) and had higher body mass index values (p=0.033). Diastolic blood pressure (DBP) (p=0.027) and carotid intima-media thickness (CIMT) values (p=0.006) were also higher in the patient group. In 24% (n=6) of CAH patients, 24-h ambulatory BP monitoring showed arterial hypertension. CIMT was significantly higher in the hypertensive patients than in those with no hypertension (p=0.013). Twenty percent (n=5) of CAH patients had nocturnal hypertension. CIMT was significantly greater in the nocturnal hypertensive group (p=0.02). Mean systolic BP (SBP) and DBP dipping were significantly different in the CAH patients (p<0.001). CIMT correlated negatively with DBP dipping (r=-0632, p=0.037) in these patients.

**Conclusion:**

These results provide additional evidence for the presence of subclinical cardiovascular disease in classical CAH patients and its relationship with hypertension.

## INTRODUCTION

Congenital adrenal hyperplasia (CAH) causes adrenal androgen excess and a variable degree of cortisol and aldosterone deficiency. More than 95% of the cases are due to 21-hydroxylase deficiency (21-OHD) (1). There is increasing evidence that patients with classical CAH have multiple vascular risk factors and that they are at increased risk for cardiovascular disease in adulthood. Children and adolescents with CAH are also at greater risk for increased fasting serum insulin levels and insulin resistance (IR) (2). Monitored measurements show that these patients also manifest elevated 24-h ambulatory blood pressure (BP) (3).

The findings cited above emphasize the importance of evaluating the development of vascular and metabolic changes in children and adolescent patients with classical CAH. Based on this background, we designed a clinical study aimed at evaluating the metabolic and cardiovascular profile of a group of children with CAH.

## METHODS

This study was performed retrospectively between August 2011 and August 2012 at the Necmettin Erbakan University, Konya, Turkey. The study was approved by the local ethics committee. We included 25 children and adolescents (16 females, 9 males) aged between 5 and 15 years with salt-wasting (SW) 21-OHD who were already being followed by the Pediatric Endocrinology Outpatient Clinic. All patients belonged to the SW form of CAH and they had been diagnosed in the neonatal period (mean age 12 days). Clinical and hormonal data of the CAH patients are presented in Table 1. Twenty-five age- and sex-matched children and adolescents who presented to the outpatient clinics of the Departments of Pediatric Endocrinology were recruited into the study as a control group. The control group had no chronic or endocrinologic disease and their weight and height percentiles were normal.

The diagnosis of 21-OHD classical CAH was confirmed by elevated serum 17-hydroxyprogesterone (17-OHP) levels and adrenocorticotropic hormone (ACTH) stimulation test. Presence of a SW state was confirmed by the findings of hyponatraemia, hyperkalaemia, elevated plasma renin activity and reduced serum aldosterone concentration. All of the SW patients were diagnosed through detection of virilization of the genitalia in girls at birth and raised renin levels or acute salt loss in the boys. All patients had been treated from the time of diagnosis. Patients whose mothers had received antenatal dexamethasone treatment were excluded from the study. Karyotype results were concordant with the sex of assignment and rearing in all patients.

All patients received replacement therapy in the form of oral hydrocortisone (15-18 mg/m2 body surface area/day) given twice or thrice daily and 9 α-fludrocortisone (100-150 μg/m2 body surface area/day) given once or twice daily. Therapy was monitored by regular assessment of clinical and laboratory data in accordance with current guidelines (4). A single-stage clitoroplasty, vaginoplasty and labiaplasty operation was performed in all female CAH patients. During follow-up, no complications such as fistulas or vaginal strictures were noted and all patients had a good genital cosmetic appearance.

The mean duration of follow-up was 7.8±3.3 years. Time of onset of puberty (defined by the onset of breast development in females and increased testicular volumes in males) was 9.8±1.7 years for girls and 12.6±1.6 years for boys. Menarche occurred spontaneously in all female patients at an average age of 12.6±1.2 years.

At the time of the analysis, none of patients showed any signs of an acute or chronic disease, nor were they receiving lipid-lowering therapy or any other medication. There was no reported history of parental hypertension. Children with any history of alcohol intake or smoking were excluded from the study.

Anthropometric data were obtained by trained research personnel. Height was measured using a wall-mounted stadiometer and weight was determined using a balance scale. Body mass index (BMI) was calculated as the ratio of weight (kg) to the square of height (m2). Hip circumference and waist circumference were measured and recorded. Pubertal staging of the patients was done according to Tanner staging (5).

### Biochemical Evaluation

Fasting blood samples for determination of serum glucose, insulin, lipid parameters, sodium and potassium were taken in the morning, between 08:00 and 09:00 h, following an overnight fast, from all patients and from all controls. Serum levels of 17-OHP, plasma ACTH and renin were measured only in CAH patients. Glucose, triglycerides, total cholesterol and high-density lipoprotein (HDL) concentrations were determined using an enzymatic colorimetric method, while chemiluminescence method was applied to assess serum insulin levels. 17-OHP and ACTH were measured using enzyme-linked immunosorbent assay and direct renin was measured by immunoradiometric method.

Hyperlipidemia was defined as a serum lipid level higher than 95% for age and sex and/or receiving lipid-lowering therapy (6). The homeostasis model assessment of IR (HOMA-IR) was used as an estimate of IR and was calculated as fasting insulin concentration (μU/mL) X fasting glucose concentration (mmol/L)/22.5 (7). IR was defined as HOMA-IR of greater than 3.16 according to our previously published data (8).

### Blood Pressure Measurements

Blood pressure measurements were performed 3 times in succession at 1-minute intervals using a mercury sphygmomanometer after the subject had rested for at least 10 minutes. The mean of the 3 measurements was recorded. Hypertension was defined as a BP value equal to or greater than the 95th percentile for age, sex and height (9). The subjects also underwent a 24-h ambulatory BP recording using the Spacelabs 90207 ambulatory monitor (Spacelabs Medical, Issaquah, WA). The BP system was programmed to measure every 20 min, from 06:00 to 22:00 hours and every 30 min, from 22:00 to 06:00. Self-reported sleep-wake times were used to divide 24-h ambulatory BP monitoring data into daytime and nocturnal periods. Ambulatory hypertension was defined as an average ambulatory systolic BP (SBP) or diastolic BP (SBP) for the measurement period was ≥95th percentile according to normative values for 24-h ambulatory BP (10). BP load was defined as the percentage of readings for a given period that exceeded the 95th percentile for that individual. Percent dipping was calculated for both average SBP and DBP using the following formula: [(daytime BP - nocturnal BP) /daytime BP] X 100. Each subject was categorized as a “dipper” (decrease in average SBP and DBP ≥10% during sleep) or a “nondipper” (decrease <10%) (11).

### Carotid Artery Ultrasonography

Longitudinal images of the common carotid artery were obtained by combined 2D-mode and color Doppler examinations. Carotid intima-media thickness (CIMT) was measured using the electronic calipers of the machines, as previously described (12). On a longitudinal, echocardiographic image of the carotid artery, the posterior wall of the carotid artery was displayed as two bright white lines separated by a hypoechogenic space. The CIMT was assessed at the far wall as the distance between the interface of the lumen and intima (first echogenic line) and the interface between the media and adventitia (second echogenic line). The mean CIMT was calculated for each child as the average of three consecutive measurements of the maximum far wall thickness obtained from the common carotid artery 10 mm below the carotid bulb.

### Statistical Analysis

For statistical analysis, the SPSS (version 16.0) package program was used. Categorical parameters are given as %. Continuous variables (age, BMI, body weight, height, lipid panel, blood glucose, SBP, DBP and CIMT are shown with mean ± standard deviation. Differences between mean values were assessed using the student’s t-test. Statistical correlation was assessed using the Pearson test (r). A p-value <0.05 was considered statistically significant.

## RESULTS

The characteristics of the study population are shown in Table 2. The groups were matched for age, gender and body size.

Mean daily hydrocortisone and 9 α-fludrocortisone replacement doses were 17.03±5.4 mg/m2 body surface area and 120±24 μg/m2 body surface area, respectively. Doses were similar in the two sexes. There was no relationship between hydrocortisone and 9 α-fludrocortisone dose expressed in terms of anthropometric and metabolic variables.

No difference was found between CAH patients and control subjects for age, weight, waist/hip ratio, or glucose, insulin, total cholesterol, low-density lipoprotein (LDL)-cholesterol triglyceride, HDL-cholesterol levels and SBP values. However, CAH patients were shorter than the controls (p=0.001) and had a higher BMI (p=0.033), a higher DBP (p=0.027) and a higher CIMT (p=0.006). Twelve percent (n=3) of the CAH patients had dyslipidemia.

While mean fasting glucose, fasting insulin and HOMA-IR index were similar in the CAH group and control subjects, 16% (n=4) of the CAH patients had IR.

Of the CAH patients, 8 (32%) were diagnosed to be hypertensive using casual BP readings, 6 (24%) patients had daytime or nighttime hypertension (hypertensive group) and 19 (76%) patients showed normal BP levels (non-hypertensive group) when classified by 24-h ambulatory BP monitoring. There were no significant differences between the hypertensive and non-hypertensive CAH patients with regard to their anthropometric and metabolic variables. However, CIMT was significantly higher in the hypertensive group than in the non-hypertensive group (0.0601±0.019 mm vs. 0.083±0.005, p=0.013).

The CAH patients were divided into two groups according to their 24-h ambulatory BP monitoring: those who had hypertension only during the night [nocturnal hypertensive group (n=5, 20%)] and those with non-hypertensive BP levels [non-hypertensive group (n=20)]. CIMT was significantly greater in the nocturnal hypertensive group (0.0758±0.012) than in the non-hypertensive group (0.0417±0.007) (p=0.02). No difference was found between the two groups for any other anthropometric or metabolic variables. Mean SBP and DBP dipping were significantly different in CAH patients as compared to the controls (p<0.001). Five (20%) of the CAH patients had impaired SBP dipping and 4 (16%) had impaired DBP dipping.

Cardiovascular parameters of study population are given in Table 3. CIMT was negatively correlated with DBP dipping (r=-0632, p=0.037) in the CAH patients. We also grouped these patients according to the degree of nocturnal dipping; no difference was found between the two groups for the anthropometric and metabolic variables.

Table 4 shows the correlations between CIMT and other cardiovascular risk factors in children with CAH.

## DISCUSSION

We studied cardiovascular and metabolic risk profiles in a cohort of children and adolescents with classical CAH due to 21-OHD. Our findings show some indications of increased cardiovascular and metabolic risk in this population, consistent with previous studies (13,14,15).

Studies have demonstrated an increased prevalence of obesity, hypertension, dyslipidemia, carbohydrate metabolism disorders and IR in CAH patients (2,3). Obesity and IR, both common findings in CAH, are known to be associated with dyslipidemia (13). To date, only a few studies have reported lipid profiles in CAH patients (13,16). The recent study by Zimmermann et al (16) showed that despite apparently normal routinely assessed lipid parameters, CAH patients could have discrete alterations in lipid metabolism characterized by an elevation in concentrations of cholesterol in the small-dense LDL subfractions (sd-LDL). Such alterations might be an additional metabolic risk predisposing the patient to an accelerated atherogenesis. Our results showed lipid profile alterations in 12% of patients.

Literature data suggest that both classic and non-classic CAH patients exhibit a higher IR as determined by HOMA-IR index (2,17). This appears to be in line with our findings, since we found IR in 16% of our patients. The cause of IR in classical CAH is its association with long-standing adrenomedullary hypofunction and decrease in catecholamine levels leading to a loss of inhibition of insulin secretion (2). In addition, periods of iatrogenic hypercortisolism may further contribute to the IR (18). In our CAH patients, the hydrocortisone dose range was within recommended guidelines (4) and comparable to other reported cohorts (3).

Increased CIMT is significantly related to known cardiovascular risk factors and to carotid plaque, a more advanced atherosclerotic lesion (19). CIMT could be a surrogate marker for cerebral and coronary events and imparts prognostic information independent of traditional cardiovascular risk factors (20).

Our study provides evidence showing a high rate of increased CIMT in CAH patients. This finding is in line with an Italian study (20) which demonstrated reduced insulin sensitivity and increased CIMT independently of cumulative glucocorticoid doses and androgen levels.

Wasniewska et al (21) also found higher CIMT values in adolescent patients with classical and non-classic CAH. In their study, CIMT was positively associated with triglycerides and triglyceride/HDL ratio. Conversely, in our study, lipid parameters were not found to be a relevant factor for CIMT. It is possible that the difference between the findings of Wasniewska et al (21) and our study is due to differences in the age range and in the level of metabolic control of the patients in the two studies.

There is minimal data on hypertension in CAH patients. However, the tendency towards an increased prevalence of hypertension has been mentioned in some reports (13,14). In CAH patients, there are several factors, primarily related to glucocorticoid and mineralocorticoid deficiency or to overtreatment, that may interfere with the physiological control of BP (22). Excessively elevated glucocorticoid levels also affect the mineralocorticoid receptor (23). Moreover, there is increasing evidence that adrenomedullar function is altered in CAH patients (24), which might have a negative effect on the sympathogenic regulation of BP in these patients. Preliminary data on 24-h BP profiles measured in a clinical setting suggested an increased risk of hypertension (3).

We observed a relatively higher prevalence of hypertension in our patients. Using 24-h ambulatory BP monitoring allowed us to show blunted diastolic BP and systolic BP dipping. Dipping of BP in the night is a normal physiological change that can be blunted by cardiovascular risk factors and severe hypertension (25).

In our patients, the 24-h ambulatory BP monitoring revealed a loss of nocturnal reduction in BP suggestive of presence of arterial hypertension in classical CAH patients otherwise considered normotensive by single ambulatory BP evaluation.

Our results demonstrated an increased CIMT in the CAH patients, a finding which was accompanied by nocturnal hypertension and impaired SBP and DBP dipping. These findings suggest that nocturnal hypertension and impaired nocturnal BP dipping may influence the development and progression of atherosclerosis in CAH patients. As hypertension is an important risk factor for cardiovascular complications, it is important that classical CAH patients should undergo 24-h ambulatory BP monitoring to detect the presence of hypertension that would be missed by a single BP measurement.

The main limitation of this study is its limited size and difficulties in assessing the impact of steroid treatment. The type of steroid administered and their cumulative lifetime doses were not available and we used the dose of the present steroid used in the calculations.

This study enabled us to assess simultaneously several cardiometabolic risk factors in patients with classical CAH. Our study also showed relationships between hypertension and nocturnal hypertension, CIMT and SBP dipping, findings which may be predictive of the degree of atherosclerosis in children with CAH. Our data underline the importance of monitoring BP in children and adolescents with classical CAH. To provide early detection and management of risk factors for macrovascular complications, 24-h ambulatory BP monitoring should be considered in children and adolescents with classical CAH.

## Figures and Tables

**Table 1:**
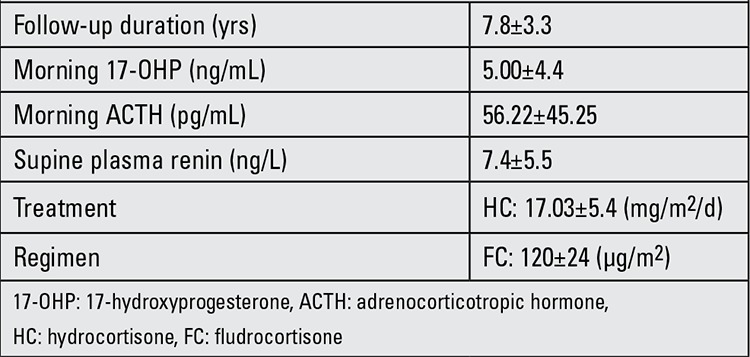
Clinical and hormonal data of the congenital adrenal hyperplasia (CAH) patients at the time of their inclusion in the study

**Table 2:**
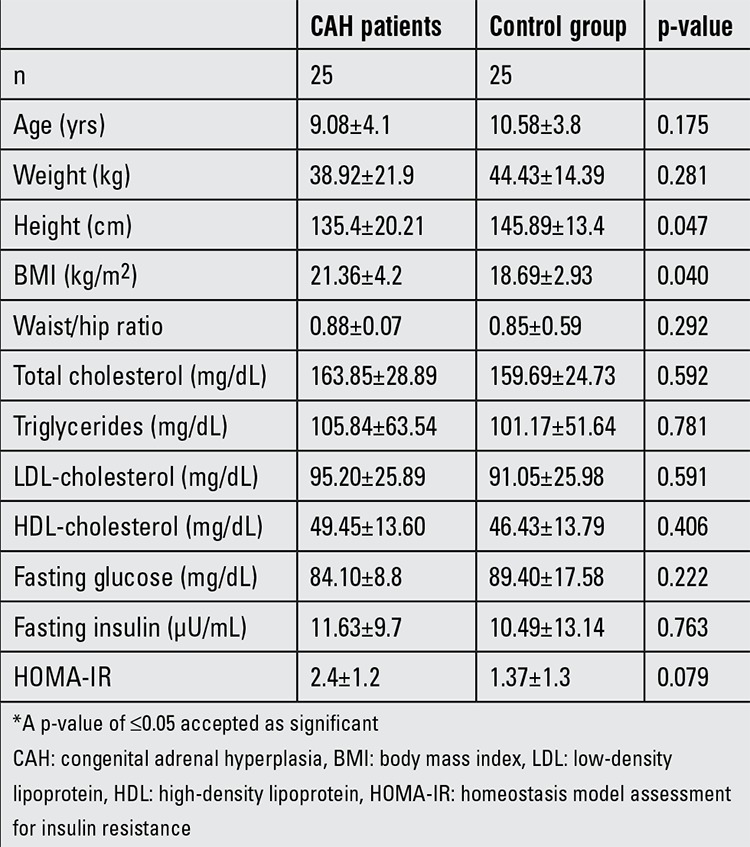
Clinical characteristics of study population

**Table 3:**
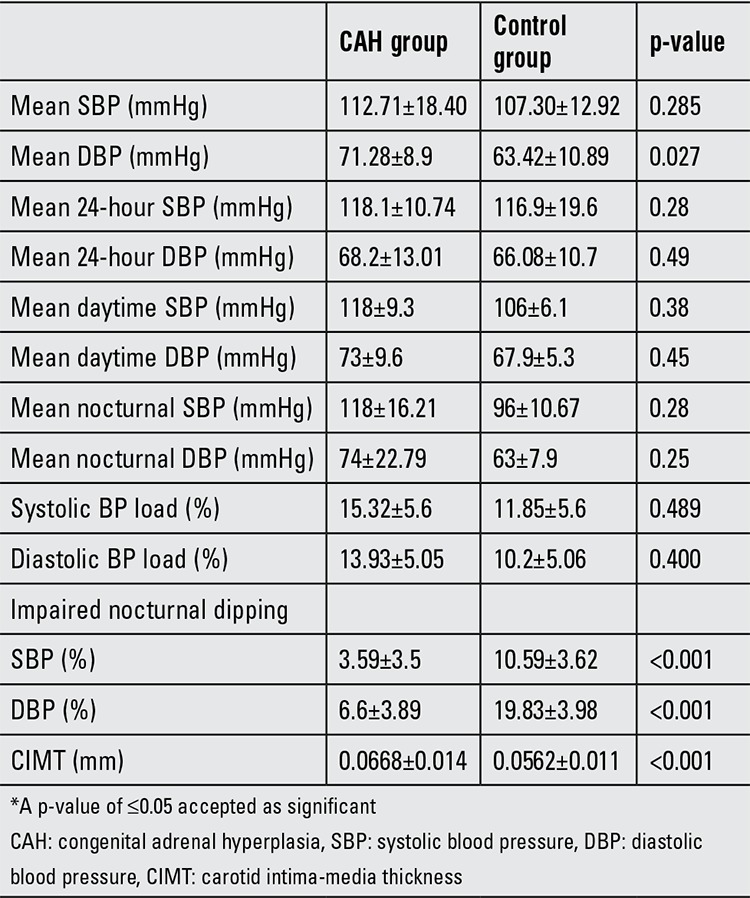
Cardiovascular parameters of the study population

**Table 4:**
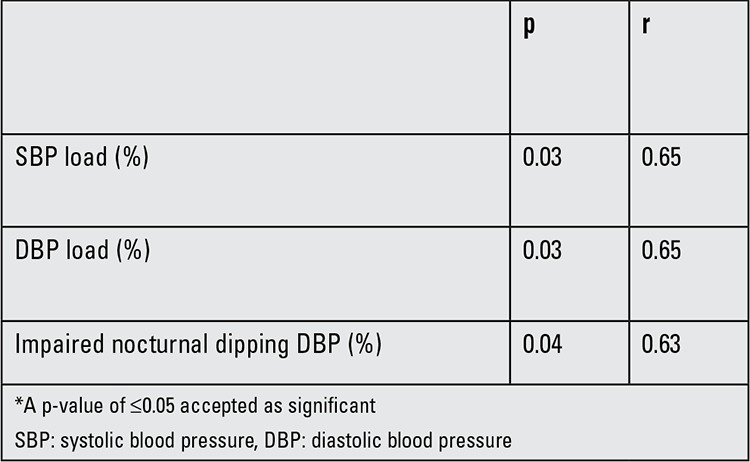
Significant correlations between carotid intima-media thickness (CIMT) and other cardiovascular risk factors in children with congenital adrenal hyperplasia (CAH)
